# A Multi-Stage Bioprocess for the Expansion of Rodent Skin-Derived Schwann Cells in Computer-Controlled Bioreactors

**DOI:** 10.3390/ijms24065152

**Published:** 2023-03-08

**Authors:** Tylor Walsh, Brett Abraham, Tak-Ho Chu, Jeff Biernaskie, Rajiv Midha, Michael S. Kallos

**Affiliations:** 1 Biomedical Engineering Graduate Program, University of Calgary, 2500 University Drive NW, Calgary, AB T2N 1N4, Canada; 2 Pharmaceutical Production Research Facility, University of Calgary, 2500 University Drive NW, Calgary, AB T2N 1N4, Canada; 3 Department of Biomedical Engineering, Schulich School of Engineering, University of Calgary, 2500 University Drive NW, Calgary, AB T2N 1N4, Canada; 4 Hotchkiss Brain Institute, University of Calgary, 3330 Hospital Dr NW, Calgary, AB T2N 4N1, Canada; 5 Department of Clinical Neurosciences, Cumming School of Medicine, University of Calgary, 3330 Hospital Dr NW, Calgary, AB T2N 4N1, Canada; 6 Faculty of Veterinary Medicine, University of Calgary, 3280 Hospital Dr NW, Calgary, AB T2N 4Z6, Canada; 7 Alberta Children’s Hospital Research Institute, University of Calgary, 2500 University Drive NW, Calgary, AB T2N 1N4, Canada

**Keywords:** Schwann cell, bioreactor, bioprocess, scale-up, nerve repair

## Abstract

Regenerative therapies for the treatment of peripheral nerve and spinal cord injuries can require hundreds of millions of autologous cells. Current treatments involve the harvest of Schwann cells (SCs) from nerves; however, this is an invasive procedure. Therefore, a promising alternative is using skin-derived Schwann cells (Sk-SCs), in which between 3–5 million cells can be harvested from a standard skin biopsy. However, traditional static planar culture is still inefficient at expanding cells to clinically relevant numbers. As a result, bioreactors can be used to develop reproducible bioprocesses for the large-scale expansion of therapeutic cells. Here, we present a proof-of-concept SC manufacturing bioprocess using rat Sk-SCs. With this integrated process, we were able to simulate a feasible bioprocess, taking into consideration the harvest and shipment of cells to a production facility, the generation of the final cell product, and the cryopreservation and shipment of cells back to the clinic and patient. This process started with 3 million cells and inoculated and expanded them to over 200 million cells in 6 days. Following the harvest and post-harvest cryopreservation and thaw, we were able to maintain 150 million viable cells that exhibited a characteristic Schwann cell phenotype throughout each step of the process. This process led to a 50-fold expansion, producing a clinically relevant number of cells in a 500 mL bioreactor in just 1 week, which is a dramatic improvement over current methods of expansion.

## 1. Introduction

Cell therapy is considered a promising treatment of spinal cord and peripheral nerve injuries [1]. Amongst many proposed cell types, Schwann cells (SCs) are one of the best candidates for nerve repair due to their inherent abilities to promote axon regeneration and remyelination [2,3]. Following nerve injury, SCs distal to the lesion dedifferentiate and adopt a repair phenotype which initiates a cascade of cellular reprogramming. These specialized repair SCs release growth factors and cytokines to support axon regeneration and recruit immune cells to further modulate repair [4,5]. Recent studies in human SCs demonstrated that they also expressed high level of HLA genes which suggest its immunomodulatory role as an antigen presenting cells [6,7]. These intrinsic properties of SCs are instrumental to nervous system tissue repair. 

A large amount of pre-clinical evidence supports the addition of SCs to improve functional recovery after spinal cord and peripheral nerve injuries [3,5,8,9,10,11]. After a comprehensive analysis of the literature, Hosseini et al. concluded that SC transplantation leads to a moderate motor function recovery after spinal cord injuries [3], with immediate transplantation of SCs leading to twice as much recovery than delayed transplantation or sham control. Similar results were reported with the use of mesenchymal-derived and skin-derived SCs in spinal cord injuries [12,13]. Not surprisingly, the benefits of SC transplantation seem to be more robust in the peripheral nervous system than in the spinal cord as demonstrated in many promising pre-clinical experimental studies [14,15,16,17,18,19,20] augmented with nerve-derived autologous SCs [21]. 

The current procedure for harvesting SCs requires surgically removing a segment of peripheral nerve, dissociating the tissue to isolate cells, and expanding the Schwann cells in vitro. This method is invasive and inefficient due to individual donor variations [22], and can cause additional comorbidity to the harvesting site. Alternatively, Schwann cells can be sourced from skin-derived precursors, isolated from small skin biopsies. These cells can then be differentiated in vitro into Sk-SCs and have been demonstrated to be quite effective at nerve and spinal cord regeneration [3,11,19,20,23,24,25]. These functionally effective cells, derived from a minimally invasive biopsy [7,26] make an ideal candidate for autologous cellular therapy for nerve injury repair.

Nevertheless, a major consideration for any cell therapy is still the total number of cells required for treatment, a value which remains ambiguous for SC therapy [27,28,29,30]. The optimal dosing is likely dependent on the type and size of lesion, type of cell to be administered, their intrinsic properties such as immunogenicity and thus survival of the transplanted cells, migration ability and efficacy of the cells to perform their purported functions. In a recent review article, the authors found that the number of transplanted cells ranged from 4 × 10^5^ to 2.2 × 10^7^ cells per kilogram of body mass [3]. Therefore, in vitro expansion of cells is typically required before clinical application [31]. Almost all cellular based therapies begin as lab-scale static cultures. In static culture most culture variables are not controlled, as controlling, monitoring, and evaluating the impact of key parameters on target cell output and productivity are difficult [32]. This can lead to large batch-to-batch variations and a non-homogeneous cell product that limits the feasibility of clinical translation.

To overcome these limitations, in this work we employ engineering principles and approaches to the scale-up of SC production using DASGIP computer-controlled bioreactors with the aim of efficiently and reproducibly generating clinically relevant numbers of SCs. We performed a proof-of-concept pilot run for the expansion of adult rat Sk-SCs in biological duplicate. Our aim was to simulate an autologous clinical process where we started our expansion process with 3 million cells, a number which reflects a conservative estimate based on what can be achieved from a single biopsy [7]. These cells were then inoculated directly into 500 mL bioreactors on Cytodex 3 microcarriers and expanded for 6 days before collecting the cell product using in-vessel harvesting. This process resulted in a clinically relevant number of cells, of a characteristic phenotype, generated in a tightly controlled and reproducible bioprocess. We also examined the survival of cells after freezing for storage/shipment to gauge the possibility of off-site handling of cells from a potential cell culture facility to the operation room.

## 2. Results

### 2.1. Pre-Expansion Cryopreservation

To model a clinical process, Sk-SCs were thawed in static 100 mm dishes and cultured for 4 days for two subsequent passages. After this initial expansion, the skin-SCs were harvested, counted, and cryopreserved in SC freezing medium. The harvested cells were divided into two vials and frozen at a density of 3.2 × 10^6^ cells/mL in 1 mL of freezing medium. Each vial was used to seed an independent bioreactor run. The cells were then held at −80 °C for 24 h and then placed in the vapor phase of liquid nitrogen for 7 days.

**Figure 1:**
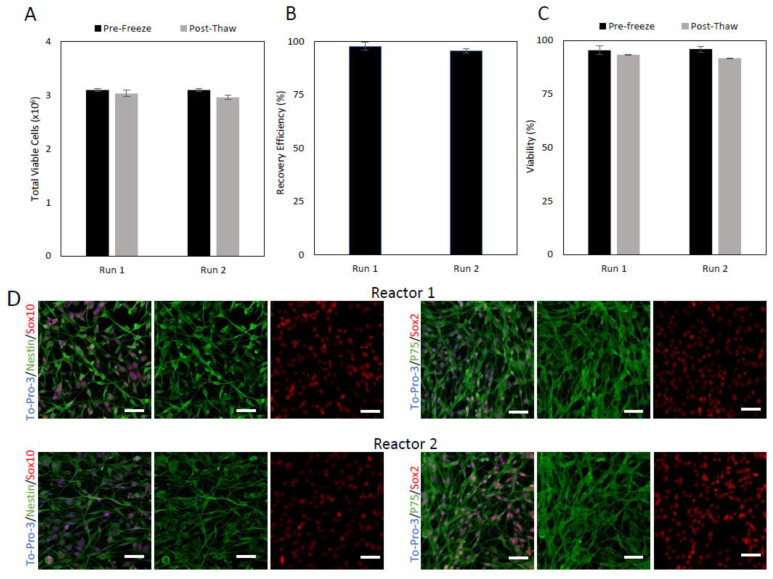
Total viable cell number, percent viability, and recovery efficiency of adult rat skin-derived Schwann cells (Sk-SCs), before and after cryopreservation. Sk-SCs were static expanded for 2 passages before being harvested and cryopreserved for 7 days to represent the shipping and storage involved in therapy. Following cryopreservation, cells were thawed and counted to achieve the total viable cells (**A**), the overall recovery efficiency (**B**), and the viability, before and after for cryopreservation (**C**). (**D**) Immunophenotype of pre-expansion Sk-SCs. The starting Sk-SCs were stained for expression of characteristic markers Sox10, Sox2, P75, and nestin before expansion in bioreactors. Scale bars represent 200 μm. Error bars represent one standard deviation of the mean.

After 7 days in the liquid nitrogen, the two runs were thawed and counted to obtain the recovery efficiency and viability prior to direct inoculation into the bioreactor (Figure 1). Of the 3.2 × 10^6^ viable cells that were frozen for runs 1 and 2, 3.03 × 10^6^ and 2.96 × 10^6^ viable cells were recovered post-thaw, respectively. This resulted in viable cell recovery efficiencies of 97.8% for run 1 and 95.6% for run 2.

After being thawed, the cells for the 2 runs were plated in chamber slides, expanded for 24 h then fixed and stained with classic SC transcription factors Sox10 and Sox2, and cytoplasmic markers nestin and p75 to demonstrate that the SCs maintained a characteristic phenotype through the cryopreservation process and for the initiation of the bioreactor expansion (Figure 1). The cells from both runs expressed each of the markers and demonstrated good co-staining between Sox10-Nestin, and Sox2-P75. The morphology of the cells was consistent between each run and comparable to our prior work with Sk-SCs [23].

### 2.2. Inoculation

**Figure 2:**
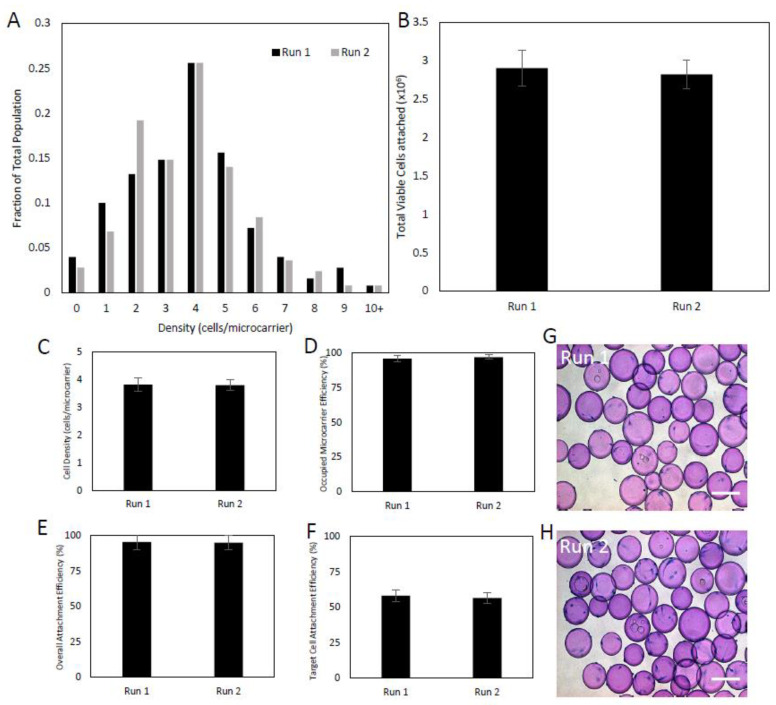
Attachment and distribution of Sk-SCs on Cytodex 3 microcarriers. Two independently isolated and cryopreserved Sk-SC samples were thawed into 500 mL DASGIP bioreactors running with intermittent agitation for the first 24 h (four 5-min agitation periods/hour at 40 rpm for 24 h). After 24 h, samples were taken to obtain the distribution (**A**), total viable attached cells (**B**), cell density (**C**), number of microcarriers with at least 1 cell (**D**), the overall attachment efficiency (**E**), and number of microcarriers achieving the target loading (**F**). (**G**,**H**) Photomicrographs of microcarriers 24 h after inoculation for runs 1 and 2, respectively. Scale bars represent 200 μm. Error bars represent one standard deviation of the mean.

Following thawing, the two runs were seeded into 500 mL computer-controlled bioreactors with a target seeding density of 4 cells/microcarrier. Each run had a wide cell-to-microcarrier distribution ranging between 0 cells/microcarrier to 10 cells/microcarrier, with a standard deviation of 2 cells/microcarrier (Figure 2A). Efficient cell-microcarrier attachment was also observed after 24 h. Of the 3 × 10^6^ cells inoculated into the bioreactor, run 1 yielded a total of 2.9 × 10^6^ cells attached, and the number was slightly lower for run 2, with 2.8 × 10^6^ cells attached to the microcarriers (Figure 2B). Both runs exhibited an average cell density of 3.8 cells/microcarriers, with 96.0% and 97.2% of microcarriers yielding at least one cell for runs 1 and 2, respectively (Figure 2C,D). The overall attachment efficiency of the inoculated cells was 95.7% for run 1 and 94.8% for run 2 (Figure 2E). Each of the runs showed greater than the predicted 51% of microcarriers achieving the target loading, with run 1 at 58.0% and run 2 at 56.4% (Figure 2F). Overall, each run displayed predictable inoculation efficiencies, and the inoculation process was found to be robust and reproducible between the two runs.

### 2.3. Expansion

**Figure 3:**
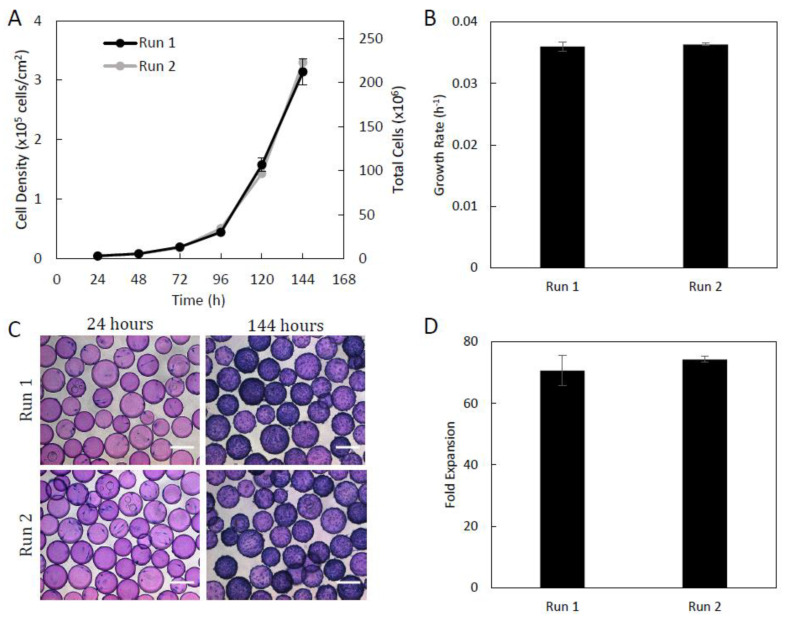
Growth kinetics of Sk-SCs expanded in 500 mL DASGIP bioreactors. Daily samples were taken over the culture duration to generate growth curves (**A**) and to calculate the growth rate (**B**) and fold expansion (**D**). (**C**) Photomicrographs of cell-laden microcarriers after 24 and 144 h of culture. Scale bars represent 200 μm. Error bars represent one standard deviation of the mean.

The culture of Sk-SCs in computer-controlled DASGIP bioreactors lead to both runs reaching similar cell densities over the duration of the culture (Figure 3). Run 1 was able to reach a maximum cell density of 3.14 × 10^5^ cells/cm^2^ and run 2 reached a cell density of 3.3 × 10^5^ cells/cm^2^, which corresponds to 2.02 × 10^8^ and 2.21 × 10^8^ total cells in each bioreactor, respectively (Figure 3A). Photomicrographs from culture day 6 clearly show confluent microcarriers with densely packed cells (Figure 3C). For this reason, Day 6 was chosen for the end of the growth curve to ensure that the cells did not become overly confluent, and therefore detach from the microcarriers, or agglomerate to form microcarrier clumps. Microcarrier aggregation is typically considered a concern for cell therapy bioprocesses and should be avoided to ensure a homogeneous population of high-quality cells [33].

**Figure 4:**
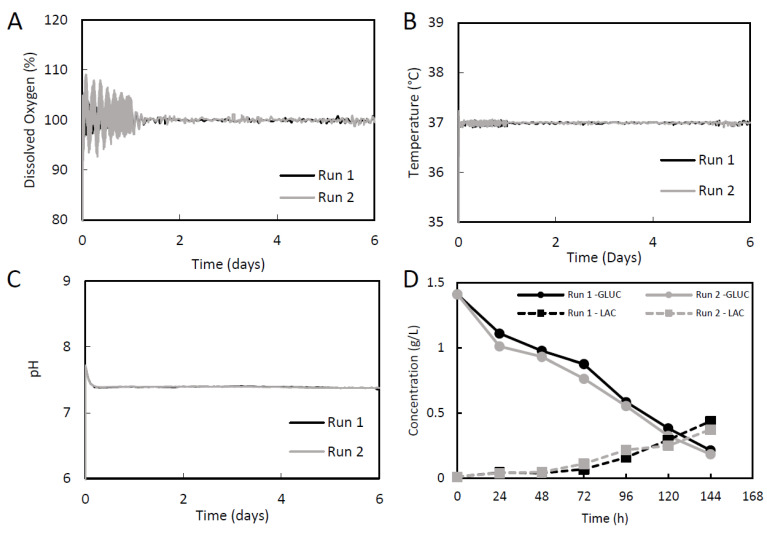
Process parameters and metabolism of Sk-SCs expanded in 500 mL DASGIP bioreactors. (**A**) Dissolved oxygen of bioreactor culture medium. Dissolved oxygen was set to 100%, corresponding to atmospheric oxygen saturation (21%). (**B**) Bioreactor temperature was set to 37 °C. (**C**) Bioreactor pH was set to 7.4. (**D**) Metabolite analysis of glucose (GLUC) and lactate (LAC). Samples were measured daily using a YSI metabolite analyzer.

Under the controlled growth parameters, the Sk-SCs in each bioreactor were able to achieve high growth rates and fold expansions (Figure 3). Run 1 had a growth rate of 0.0360 h^−1^, a doubling time of 19.26 h, and a fold expansion of 70.7. Run 2 had a growth rate of 0.0364 h^−1^, a doubling time of 19.15 h, and a fold expansion of 74.3 (Figure 3B,D). Figure 4 shows that in the 500 mL computer controlled DASGIP bioreactors, the dissolved oxygen, pH, and temperature were well controlled within the set points (100% DO, 7.4 pH, and 37 °C) during the expansion of the skin-SCs for the two independent runs. The large fluctuations in the dissolved oxygen during the first 24 h was due to the intermittent agitation.

The use of a relatively low microcarrier density (0.5 g/L) in the bioreactors allowed for the operation of an unfed batch culture. Based on these surface area densities, each bioreactor generated just over 4 × 10^5^ cells/mL. Because of the lower cell density in this process, there was no need for feeding the skin-SCs, as shown through metabolite analysis. There was a steady decline in the concentration of glucose from 1.4 g/L to 0.2 g/L over the duration of the culture, but it was not depleted (Figure 4D). If more cells were present, or if the culture was carried out for longer time, feeding would become necessary due to glucose depletion. Lactate production was shown to be quite low in this Sk-SCs bioprocess. On culture day 6, the lactate concentration was 0.45 g/L for run 1 and 0.37 g/L for run 2, suggesting that removal of lactate by-products through a feeding protocol is similarly unnecessary. Overall, the metabolite analysis suggests that there is no need to feed these reactors at the cell concentrations achieved during this process.

### 2.4. Harvest

**Figure 5:**
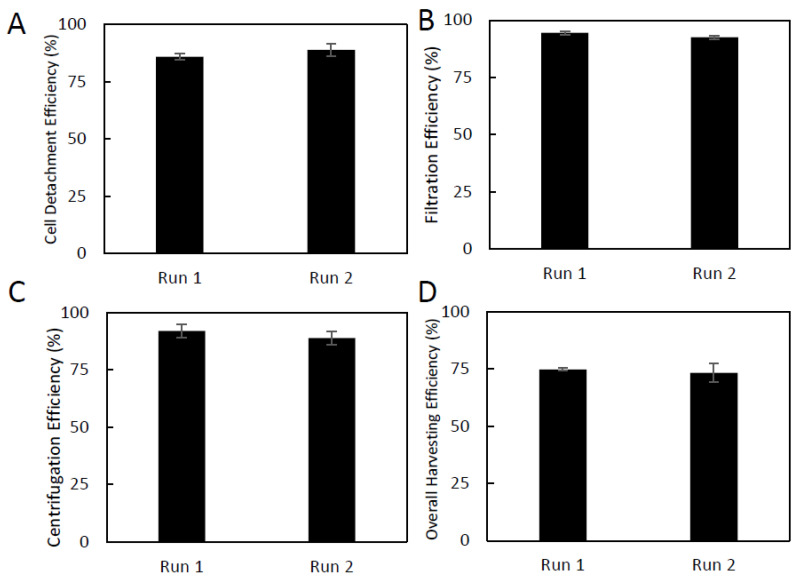

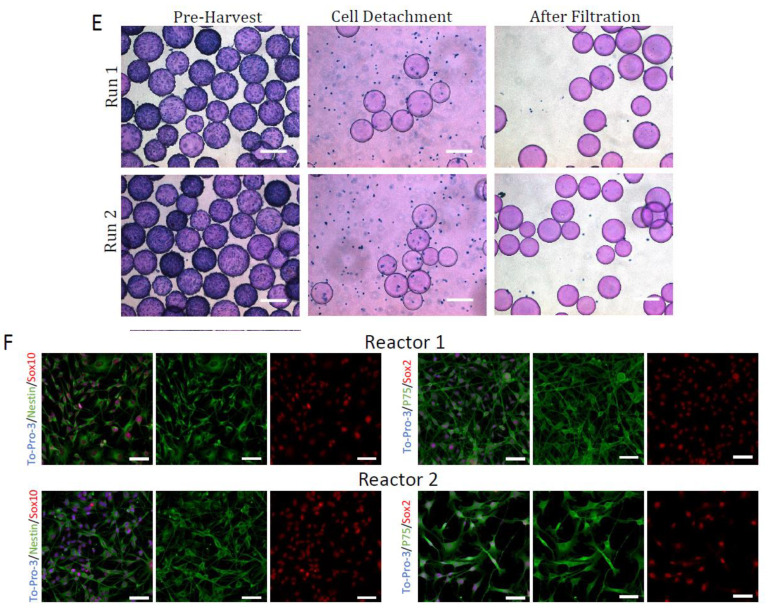
Full vessel harvest of Sk-SCs expanded in 500 mL DASGIP bioreactors, and efficiencies for each harvesting step. (**A**) Cell-microcarrier detachment efficiency, (**B**) cell filtration efficiency, (**C**) centrifugation efficiency, (**D**) overall cumulative harvest efficiency. (**E**) Photomicrographs taken before harvest, following enzymatic detachment, and after final filtering. (**F**) Immunophenotype of harvested Sk-SCS. The harvested Sk-SCs were stained for expression of characteristic markers Sox10, Sox2, P75, and nestin. Scale bars represent 200 μm. Error bars represent one standard deviation of the mean.

At each stage of the harvesting period, samples were taken for counting to determine the cell recovery for each step (Figure 5A–D). During the enzymatic in-vessel cell detachment phase, using trypsin, run 1 exhibited a viable cell detachment efficiency of 86%, and bioreactor 2 showed a detachment efficiency of 89%. After detachment, the suspension was processed through a 40 µm strainer to separate the SCs from the microcarriers, with a viable cell recovery efficiency of 94.5% for run 1 and 92.5% for run 2. These values correspond to generally acceptable recovery efficiencies, as there are several opportunities for cell loss throughout the process, including in the reactor, the pipette, the filter, or on the microcarriers. After filtration, the cell solution was centrifuged. The centrifuge step yielded a larger loss than expected, with viable cell recovery rates of 92% and 89% for runs 1 and 2, respectively. The total cell loss over all harvesting steps resulted in an overall recovery efficiency of 74.7% for run 1 and 73.3% for run 2. In general, the harvesting procedure was shown to be very reproducible between the two runs. Post-harvest Sk-SCs retained a normal growth morphology and showed excellent co-expression of both sets of characteristic markers: Sox10-Nestin and Sox2-P75 (Figure 5F). This indicates that the phenotype of the skin-SCs was not affected by the expansion or harvesting protocol.

### 2.5. Post-Expansion Cryopreservation

**Figure 6:**
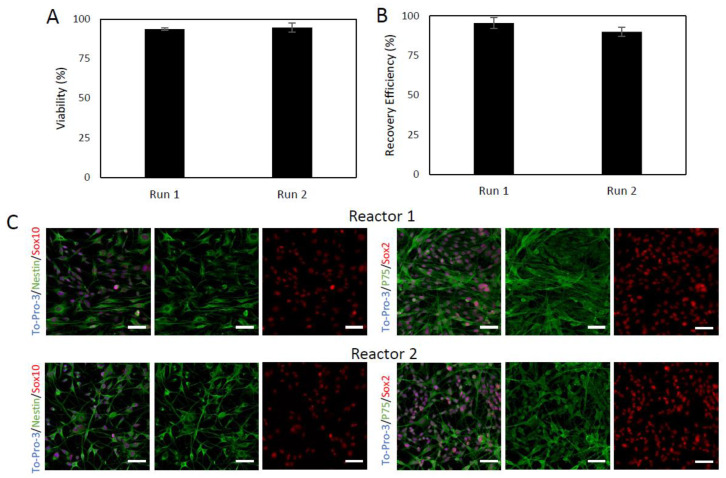
Viability and recovery efficiency following post-harvest cryopreservation of Sk-SCs expanded in 500 mL DASGIP bioreactors. Harvested Sk-SC were cryopreserved and stored in liquid nitrogen for 7 days to represent the shipping and storage requirements of a cell therapy. The cells were thawed and assessed for viability (**A**) and recovery efficiency (**B**). (**C**) Immunophenotype of post-expansion cryopreserved Sk-SCs. The post-expansion Sk-SCs were stained for expression of characteristic markers Sox10, Sox2, P75, and nestin. Scale bars represent 200 μm. Error bars represent one standard deviation of the mean.

High cell viability and the characteristic phenotype were also shown to be maintained following post-expansion cryopreservation. Following harvest on culture day 6, the cell samples were cryopreserved in freezing medium at 10 × 10^6^ cells/mL. After 7 days, the vials were thawed and the cells counted to determine cell recovery efficiency and viability (Figure 6). The viability of run 1 was 93.6%, with a recovery efficiency of 95.5%, while the skin-SCs in run 2 expressed a viability of 94.7% and a recovery efficiency of 90.1%. These recovery efficiencies are acceptably large and similar, showing good reproducibility in this cryopreservation protocol, with no significant difference between the two runs. Moreover, the phenotype of the skin-SCs was not affected by the cryopreservation protocol, as demonstrated by marker co-expression (Figure 6).

### 2.6. Overall Process

**Figure 7:**
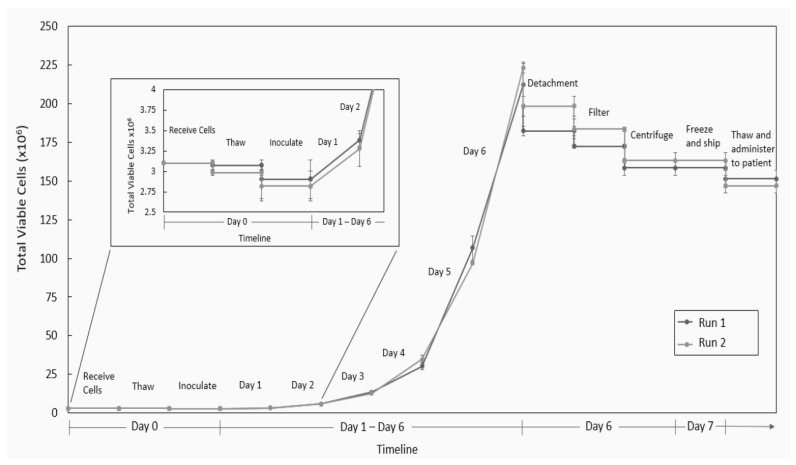
Overall process of Sk-SCs expanded in 500 mL DASGIP bioreactors. Two independent Sk-SC bioreactor runs were conducted in 500 mL DASGIP bioreactors. The cells were frozen for 7 days, after which they were thawed and directly inoculated into the bioreactor. The Sk-SCs were then expanded for 6 days, and then harvested and cryopreserved for another 7 days. The cells were then thawed and evaluated for the total cell number that could be administered to a patient.

The process started with 3 × 10^6^ skin-SC cells that were “harvested from the patient,” cryopreserved, and stored for 7 days to model shipping to a central manufacturing facility. They were then thawed directly into the bioreactor on Cytodex 3 microcarriers. The cells were cultured using inoculation conditions for 24 h, followed by expansion conditions for the next 5 days. After a total of 6 days of culture, the process produced over 2 × 10^8^ cells in each of the two 500 mL bioreactors. The cells were then harvested, and an overall recovery efficiency of approximately 74% was achieved. After harvest, the cells were cryopreserved and stored for 7 days to simulate shipment back to the patient. After thawing the frozen cells, we achieved a total of 1.5 × 10^8^ cells available for testing and administration to the patient. Figure 7 summarizes the process timeline and total cells at each stage of the process. Overall, this is a robust and efficient process that can acquire 3 million cells from a patient biopsy, expand them to 150 million (50-fold expansion), and ship them back to the clinic for patient treatment in as little as 7 days.

## 3. Discussion

The goal of this study was to simulate an autologous clinical process for Sk-SC therapy. The steps considered included receiving frozen cells from the patient/clinic, processing the cells in computer-controlled bioreactors, and finally cryopreserving the cells and shipping them back to the patient/clinic for thawing and administration. From a clinically relevant skin biopsy, using current best static culture methods ~10 × 10^6^ cells can be generated in 5–6 weeks [7,24]. These cells can then be cryopreserved and shipped to a facility for expansion. We chose to simulate the process with a basis of 3 × 10^6^ cells as a conservative estimate based on what can be achieved from a single biopsy [7]. 

The inoculation conditions (cells/microcarrier, and g/L microcarriers) were chosen based on a prediction profile developed in one of our previous studies to optimize the thaw and direct inoculation into bioreactors (unpublished data). This direct inoculation allowed for the elimination of a seed train prior to the full-scale production, reducing overall time, materials, and labor needed for each run. The inoculation efficiency and distribution of cells was found to be very high, with nearly all the cells attaching to the microcarriers. This high inoculation efficiency and even distribution of cells is essential for an efficient process. If there is poor attachment or distribution, then the available surface area and timing of the process can be negatively affected leading to poor yield of cells at the end of the process. 

The efficient and reproducible inoculation results achieved lead to high overall reproducibility in the expansion of skin-SCs between both bioreactor runs. By using bioreactors with computer-controlled set points, and maintaining each bioreactor at the same values, not only are higher growth kinetics and cell densities achieved, but there is less variability between runs compared to expansion in uncontrolled spinner flasks [34]. The culture parameters were maintained within a tight window around each set point allowing for the best conditions to expand the skin-SCs. Because of this, the process was able to reach its maximum cell density after just 6 days of culture, producing enough cells to be clinically relevant. The results of the metabolite analysis show that at these cell densities the process can be run as a batch culture, capping the medium requirements at just 500 mL. By selecting the expansion conditions to maximize cell density while minimizing the amount of reagents needed, the described bioprocess minimizes cost. 

Significant cell losses were observed across the harvesting steps of this process, indicating harvesting is an area where further improvements and optimizations will be required. The greatest losses occurred when detaching the cells from the microcarriers and separating the cells from the microcarriers. Recently, a number of responsive microcarriers have been investigated that respond to stimuli like pH and temperature, or enzymatically dissolve [35]. These microcarriers aim to limit the exposure of cells to potentially harmful harvesting enzymes through modifications to the microcarrier during the harvesting step as opposed to the cells themselves [36]. The use of dissolvable or stimulus responsive microcarriers could be used to improve harvesting of SCs in bioreactor studies. Further, in this work the separation of cells from microcarriers following in-vessel harvesting was performed by filtering the cell and microcarrier solution through a 40 µm strainer. This filtering process is only driven by gravity, and there exist commercially available filtration devices that employ a vacuum to improve filtration efficiency, such as the Sterifil Aseptic System [37] and the Steriflip unit [38] which have both been used to achieve good cell recovery and viability in Mesenchymal Stem Cell applications. Small scale vacuum filtration units such as the ones mentioned, or larger scalable units like the Harvestainer BioProcess Container [39] could be used to improve cell recovery of SCs. 

A surprisingly large cell loss was also observed during the volume reduction step. This was most likely due to the large volume being centrifuged, and the flat-bottomed bottle the cells were centrifuged in. To increase the centrifugation efficiency, the cells could be centrifuged for longer, or at a higher rate, and decreasing the brake on the centrifuge could also help. Another way to increase this efficiency would be to change the centrifuge container. Instead of using the flat bottom bottles, 500 mL conical tubes are available, this would create a tighter pellet and reduce the number of cells that are resuspended in the supernatant after centrifugation. A number of scalable and automated washing and concentrating technologies exist that have recently been reviewed [40] that could also be applied to large-scale SC bioprocessing.

The cryopreservation of cells is a very important step in any process. In this work, the methods used for both pre-expansion, and post-harvest allowed for high recovery rates and viabilities. With autologous therapies, it would be difficult, costly, and impractical to have equipment and trained operators in every hospital or clinic. Therefore, after the skin-SCs are harvested and purified, there may be a need to ship the cells to a production facility for the expansion process. Based on these results, the skin-SCs can easily be cryopreserved and shipped back and forth between the clinic and a production facility, while maintaining their Schwann cell phenotype throughout each step of the process.

The bioprocess described here can generate clinically relevant numbers in just 1 week, greatly improving the current time frame for the production of Schwann cells using conventional static expansion protocols [24,41]. This is a significant result as time is often a critical factor in the success of repairing many injuries [42]. From a clinically relevant skin biopsy, using the current best static culture methods, 9–10 × 10^6^ cells can be generated in 5–6 weeks [7]. By significantly decreasing the time to treatment, this increases the probability of successful nerve regeneration. 

One of the greatest benefits of using microcarrier bioreactor protocols for the expansion of skin-SC is that this type of process is highly flexible. Since each type of injury is different, the number of functional cells needed for efficient and effective nerve and spinal cord repair can have a broad range. Therefore, an adaptable process such as this one is highly advantageous. If a greater number of cells are needed, a static or small-scale bioreactor seed train can be developed to increase the number of starting cells, and the surface area in the reactor can be increased by adding more microcarriers. We have previously shown that by starting with 12 million cells with a microcarrier density of 2 g/L, about 667 million cells can be generated before harvest [34]. Using the overall harvesting efficiency determined in this study, by increasing the starting cell number by 4-fold we can generate an estimated 500 million cells. Bioreactors also allow for automation and a fully closed system using sterile tubing, welding, and pumps to move the components in and out of the bioreactors, reducing the probability of contamination and variability due to human interaction. 

A major unanswered question remains whether Sk-SCs retain regenerative and therapeutic function throughout a bioreactor expansion process. SCs and SKP-SCs have been demonstrated to improve neural repair through different mechanisms. Clinical trials have generated promising results using autologous human SC injections for both spinal cord injury [43] and sciatic nerve injury [21]. Further, cell-free SC derivatives including nerve grafts loaded with SKP-SC derived extracellular vesicles, and nerve grafts functionalized with SKP-SC acellular matrix, have been used to improve peripheral nerve repair in mice [44,45]. However, these studies use well-established traditional static culture methods for the expansion of SCs before in vitro or in vivo applications. It has yet to be shown whether differences in culture geometry and microenvironment between static and suspension culture impacts the functional phenotype of SCs. However, we have shown that adult rat skin-derived precursor Schwann cells retain their myelinating capabilities in an injured sciatic nerve model after expansion on microcarriers in bioreactors [23]. While these cells are from a different source, the bioreactor environment did not impact their function in vivo. In the current study, rat Sk-SCs were shown to retain characteristic immunophenotype throughout the bioreactor expansion process. This is a promising preliminary result indicating no major phenotypic alterations occurred. However, future studies will be needed to assess the therapeutic capacity of bioreactor expanded Sk-SCs through direct in vitro assays and in vivo models.

Finally, our group has recently developed a reliable protocol to harvest endogenous SCs directly from human skin [7]. This procedure circumvents the need to sacrifice healthy donor nerve to collect SCs and bypasses the need to differentiate SCs from SKPs. We have shown that these human skin-derived SCs are nearly identical to nerve-derived SCs in genomic and functional comparison. We believe that the bioprocess developed here using rodent Sk-SCs establishes the groundwork for a human skin-derived SC bioreactor process. The protocols presented here should translate well to the human skin-derived SC process which will allow for a rapid bioprocess development for human clinical application.

## 4. Materials and Methods

### 4.1. Adult Rat Skin-SC Isolation and Maintenance

Sk-SCs were isolated from rats and differentiated as previously described [26]. Once the purified Sk-SCs were obtained, they were subsequently passaged to generate a working cell bank. The cells were then thawed from this cell bank for all experiments and remained between passages 4–6. The experiments in this study were approved by the Animal Care committee at the University of Calgary and in accordance with Canadian council on animal care guidelines.

### 4.2. Pre-Expansion Cryopreservation

Rat SK-SCs were thawed and plated on poly-D-lysine (PDL)/laminin coated 100 mm culture dishes and cultured for 4 days. The cells were then passaged onto another set of PDL/laminin coated 100 mm culture dishes for an additional 4 days. On day 4, the Sk-SCs were harvested and cryopreserved at 1 × 10^6^ cells/mL in complete SC medium with 10% DMSO (Sigma Aldrich Cat# D4540-500 mL, St. Louis, MO, USA). To cryopreserve the cells, 1 mL of cells and cryopreservation medium was placed in a cryovial and cooled at 1 °C/min to −80 °C. The frozen cells were then placed in the vapor phase of liquid nitrogen. The frozen vials were stored in the liquid nitrogen for 7 days and then thawed to evaluate the viability, recovery efficiency, and inoculation efficiency of the cells.

### 4.3. Post-Expansion Cryopreservation

Following the expansion and harvest of the rat Sk-SCs on microcarriers in the bioreactors, the cells were cryopreserved in complete SC medium at 10 × 10^6^ cells/mL. To cryopreserve the cells, 1 mL of cells and cryopreservation medium was placed in a cryovial and cooled at 1 °C/min to −80 °C. The frozen cells were then placed in the vapor phase of liquid nitrogen. The frozen vials were stored in the liquid nitrogen for 7 days and then thawed to evaluate the viability and recovery efficiency.

### 4.4. Culture Media

Each culture condition was inoculated with medium from the same large batch. The SC medium was prepared fresh on the day of inoculation. To prepare the medium a 3:1 ratio of DMEM (Fisher Scientific, Cat# 11885-092, Hampton, NH, USA) and F12 (Fisher Scientific, Cat# 31765-035) was supplement with 50 ng/mL neuregulin (Fisher Scientific, Cat# 377-HB-050), 5 µM forskolin (Sigma Aldrich, Cat# F6886-10 mg), 1% N2 supplement (Fisher Scientific, Cat# 17502-048), 1% Penicillin/streptomycin (Fisher Scientific, Cat# 15140-112), and 1% fetal bovine serum (Gibco, 12483-012, Thermo Fisher Scientific, Inc., Waltham, MA, USA) [26]. The medium was then filtered using a 0.2 µm bottle top filter and incubated at 37 °C and 5% CO_2_ for a minimum of 2 h prior to inoculation with cells. 

### 4.5. Static Culture

For the static culture of the rat Sk-SCs, the cells were expanded on 100 mm culture dishes (BD Bioscience, Cat# 353003, Franklin Lakes, NJ, USA) coated with PDL (20 μg/mL) (BD Bioscience, Cat# BD354210) and laminin (4 µg/mL) (BD Bioscience, Cat# BD354232) at an inoculation density of 5000 cells/cm^2^. The dishes were incubated at 37 °C and 5% CO_2_. Cells were harvested when the confluency of the dish was 80%, typically around day 4, and either passaged into static dishes for further propagation or cryopreserved.

### 4.6. Microcarrier Preparation

Cytodex 3 microcarriers (GE Healthcare, Cat# 17-048501, Chicago, IL, USA) were weighed out in a sterile Erlenmeyer flask in the biosafety cabinet. A total of 100 mL of 1x PBS was then added to the microcarriers, ensuring that all microcarriers were washed off the side of the flask. A total of 2 drops of Tween 80 (United State Chemical Corporation) was then added to the microcarrier suspension to reduce surface tension and allow the microcarriers to settle. The microcarriers were hydrated overnight. The PBS was removed, and the microcarriers were washed 2 times with 1x PBS by adding the PBS, allowing the microcarriers to settle, then removing the PBS. After the second wash, the microcarriers were then resuspended in 1x PBS and held at 4 °C until needed. Prior to inoculation of microcarriers into the bioreactor, they were washed with DMEM and then added to the bioreactor with SC medium and allowed to incubate at 37 °C with 5% CO_2_ for at least 6 h. After the final incubations, the cells were added to the bioreactors.

### 4.7. Bioreactor Preparation

The 500 mL DASGIP bioreactor vessels were siliconized using Sigmacote. The glass vessels were washed with double distilled water and air dried. A total of 10 mL of Sigmacote (Sigma Aldrich, Cat# SL-2) was added to the vessel and swirled to coat the bottom and sides. The Sigmacote was removed, and the vessels were allowed to dry overnight. The next day, 500 mL 0.01 M PBS (pH 7.4) was added and incubated at room temperature for at least 8 h or overnight and then removed. A total of 500 mL of double distilled water was then added and incubated at room temperature for at least 8 h or overnight. The water was then removed, and the vessels were allowed to dry. The vessels were then autoclaved prior to use.

### 4.8. Bioreactor Culture

The 500 mL DASGIP bioreactor was set up by first performing off-line calibrations for the pH probe and the dissolved oxygen (DO) probe. The bioreactor vessel and probes were then autoclaved. Once the bioreactors cooled, 300 mL of Schwann cell medium was added to the bioreactor with 0.25 g of Cytodex 3 microcarriers (0.5 g/L final density) and incubated on the bench top control system for a minimum of 6 h, with the vessel temperature setpoint at 37 °C and the headspace gassing set to 5% and 21% for CO_2_ and O_2_, respectively. During this time, online calibrations for the DO and pH probes were performed. The SCs were then thawed and inoculated at a cell density of 4 cells/microcarrier with an additional 200 mL of Schwann cell medium for a total of 500 mL. An intermittent agitation flow regime was applied for the first 24 h to facilitate increased cell to microcarrier attachment, consisting of 32 cycles with 3 min on at 40 rpm, followed by 27 min off. After the first 24 h, the agitation was switched to continuous at 40 rpm. For the duration of the culture period, the set points of the controlled bioreactor were set to 37 °C, 100% DO, and pH 7.4. Each day, 2x 3 mL samples were taken for counting, as well as a 1x 0.5mL sample for the photomicrographs. The supernatant from the 3 mL samples was stored at −20 °C for metabolite analysis.

### 4.9. Metabolite Analysis

Media samples were taken daily from each condition and collected in microcentrifuge tubes. Samples were centrifuged at 3000 rpm for 3 min to remove any cells or microcarriers. The supernatant was removed, transferred to a new microcentrifuge tube, and frozen at −20 °C until analyzed. At the end of the culture period, the samples were thawed overnight at 4 °C and analyzed using a NOVA Bioprofile 100 Plus to measure the concentrations of glucose and lactate.

### 4.10. Sample Counting

To determine inoculation efficiency, 2x 3 mL samples were taken from each condition after the 24 h inoculation period and placed in a 15 mL conical tube. The microcarriers were allowed to settle before being washed 2x with 1 mL PBS. The supernatant was removed and 1 mL of 4% PFA was added to fix the cells to the microcarriers for 15 min. The PFA was removed and the microcarriers were washed 2x with 1 mL PBS, allowing the microcarriers to settle in between washes. The microcarriers were then transferred to a 6-well plate and stained with 0.5% crystal violet in methanol for visualization. The cells were then counted per microcarrier to obtain a distribution in the bioreactor. A total of 500 microcarriers were counted from each bioreactor.

For microcarrier sampling, 2x 3 mL samples were collected from the bioreactors via the in-line sampling port using sterile syringes. All microcarrier samples were allowed to settle for 5 min before rinsing with PBS. Microcarriers were then settled a second time before being lysed and stained with 1mL of 0.1M citric acid solution with 0.1% crystal violet. Samples were then incubated for 1 h at 37 °C, and the suspension was triturated 30 times to ensure the cells are lysed and the nuclei released. The released nuclei were then counted using a hemocytometer.

### 4.11. Cell Harvest

Harvesting from the 100 mm dishes was performed by first removing and discarding the supernatant culture medium. The dish was then washed twice with 5 mL PBS, and 3 mL of TrypLE Express (Life Technologies, Cat# 12605-028, Carlsbad, CA, USA) was added. The dishes were then incubated at 37 °C and 5% CO_2_ for 5 min. The dishes were periodically observed under the microscope to ensure that the cells were detached; if the cells were not detached, the dishes were tapped to dislodge the cells. The cell suspension was then removed from the dishes and placed in a conical tube. The dish was then washed twice with PBS, and the PBS was added to the conical tube. The cell suspension was then centrifuged at 1200 rpm for 5 min. The supernatant was removed, and the cells were suspended in an appropriate volume of SC medium prior to counting.

For in-vessel harvest, following the 6-day culture period, the 500 mL bioreactors were harvested using in-vessel harvesting methods. The microcarriers were allowed to settle for 5 min. They were then washed 2x with 250 mL PBS. Then, 500 mL of trypsin was added to the bioreactors. The bioreactors were then agitated at 200 rpm for a duration of 15 min. The cells, microcarriers, and medium were then filtered through a 40 µm strainer to remove the microcarriers, washed, then counted using trypan blue (Sigma Aldrich, Cat# T8154) and a hemocytometer.

### 4.12. Immunocytochemistry

The cells were then replated in chamber slides at a cell density of 20,000 cells/well and incubated for 24 h at 37 °C and 5% CO_2_. After 24 h, the supernatant was removed, and each chamber was washed 2x with 100 μL PBS. The cells were then fixed in 4% paraformaldehyde (PFA) (Mallinckrodt, Cat# CAS30525-98-4) for 15 min. The PFA was removed, and the cells were permeabilized using 0.5% Triton x-100 and 5% BSA in PBS for 1 h at room temperature. Primary antibodies, including nestin (Santa Cruz Biotechnology, Cas# SC-23927, Dallas, TX, USA), p75 (Promega Corporation, Cas# G3231, Madison, WI, USA), Sox2 (Santa Cruz Biotechnology, Cas# SC365823), and Sox10 (Santa Cruz Biotechnology, Cas# SC-17343), were added to the cells and incubated overnight at 4 °C. The next day, the supernatant was removed, and the cells were washed 2x with PBS before the addition of Alexa Fluor-conjugated secondary antibodies at a 1:200 dilution. The cells were incubated at room temperature for 2 h. The supernatant was removed, and the nuclei were counterstained with To-Pro-3 (Invitrogen, Cas# T3605, Waltham, MA, USA) at a dilution of 1:1000. The supernatant was removed, and the cells were washed 2x with PBS prior to mounting with FlourSave reagent (Calbiochem, Cat# 345789). The slides were then stored at 4 °C prior to imaging with an LSM 700 confocal microscope.

## 5. Conclusions

A bioprocess for the production of cells can be broken into upstream and downstream processes. Each of these can then be separated into individual unit operations. We have previously developed protocols for each of the necessary unit operations for the expansion of Sk-SCs on microcarriers. This included microcarrier selection, bioreactor selection, and the development of inoculation, expansion, harvesting, and cryopreservation protocols. This study took each of these individual unit operations and integrated them into a potential clinical process. We showed that we can take Sk-SCs from a clinical sized biopsy, ship them to a central facility, expand them to therapeutic numbers, cryopreserve them, and ship them “back to the patient” in as little as 7 days, greatly improving on the current time frame for spinal cord and peripheral nerve therapies. There is still work to be done in terms of closing the process, testing additional cells lines, and functional testing, but the systematic development of a fully integrated bioprocess for the production of Sk-SCs has moved therapies for peripheral nerve and spinal cord injuries one step closer to the clinic.

## Data Availability

Not applicable.
